# Role of Endoplasmic Reticulum Stress in Proinflammatory Cytokine–Mediated Inhibition of Trophoblast Invasion in Placenta-Related Complications of Pregnancy

**DOI:** 10.1016/j.ajpath.2018.10.015

**Published:** 2019-02

**Authors:** Cheuk-Lun Lee, Jan H.W. Veerbeek, Tirtha K. Rana, Bas B. van Rijn, Graham J. Burton, Hong Wa Yung

**Affiliations:** ∗ Centre for Trophoblast Research, Department of Physiology, Development and Neuroscience, University of Cambridge, Cambridge, United Kingdom; † Department of Obstetrics and Gynaecology, LKS Faculty of Medicine, The University of Hong Kong, Hong Kong; ‡ Shenzhen Key Laboratory of Fertility Regulation, Department of Obstetrics and Gynecology, The University of Hong Kong-Shenzhen Hospital, Shenzhen, China; § Department of Obstetrics and Gynaecology, Division of Woman and Baby, University Medical Center Utrecht, Utrecht University, Utrecht, the Netherlands

## Abstract

Shallow extravillous trophoblast (EVT) invasion is central to the pathophysiology of many pregnancy complications. Invasion is mediated partially by matrix metalloproteinases (MMPs). MMP-2 is highly expressed in early pregnancy. MMP activity can be regulated by proinflammatory cytokines, which also induce endoplasmic reticulum (ER) stress in other cells. We investigated whether proinflammatory cytokines regulate MMP-2 activity through ER stress response pathways in trophoblast before exploring potential regulatory mechanisms. There was increased immunoreactivity of heat shock 70-kDa protein 5, also known as 78-kDa glucose regulated protein, in cells of the placental bed, including EVTs, in cases of early-onset preeclampsia compared with normotensive controls. Treating EVT-like JEG-3 and HTR8/SVneo cells with ER stress inducers (tunicamycin and thapsigargin) suppressed *MMP2* mRNA and protein expression, secretion, and activity and reduced their invasiveness. A cocktail of proinflammatory cytokines (IL-1β, tumor necrosis factor-α, and interferon-γ) suppressed MMP-2 activity in JEG-3 cells and was accompanied by activation of the PKR-like ER kinase (PERK)–eukaryotic translation initiation factor 2A (EIF2A) arm of the ER stress pathway. Knockdown of *ATF4*, a downstream transcriptional factor of the PERK-EIF2A pathway, by small interference RNA, restored *MMP2* expression but not cellular proteins. However, suppression of EIF2A phosphorylation with a PERK inhibitor, GSK2606414, under ER stress, restored MMP-2 protein. ER stress regulates MMP-2 expression at both the transcriptional and translational levels. This study provides the first mechanistic linkage by which proinflammatory cytokines may modulate trophoblast invasion through ER stress pathways.

The invasion of extravillous trophoblast (EVT) into the decidualized endometrium is crucial in the determination of pregnancy outcome. Inadequate trophoblast invasion not only leads to implantation failure^1^ and spontaneous pregnancy loss but also results in the insufficient remodeling of spiral arteries that sits at the epicenter of the great obstetric syndromes, including idiopathic fetal growth restriction (FGR),^2^ early-onset preeclampsia,^3^, ^4^ and preterm birth.^5^, ^6^ The EVTs invade soon after implantation and complete the process around midgestation, penetrating as far as the inner one-third of the myometrium. Although many factors and biomolecules, such as transforming growth factor-β, kisspeptin, hypoxia, and the interaction with immune cells, have been proposed to regulate the invasiveness of the EVTs, their downstream effectors principally converge on a family of matrix metalloproteinase (MMP) enzymes, which break down both matrix and nonmatrix proteins.^7^, ^8^, ^9^ MMP-2 and MMP-9 are likely two key players. MMP-2 mediates trophoblast invasion during the early implantation stage up to 7 to 8 weeks of gestation, whereas MMP-9 facilitates subsequent invasion.^10^, ^11^, ^12^, ^13^ Although the regulation of MMP activity has been widely studied, the mechanisms remain largely unknown.

MMPs are controlled at multiple levels. Transcriptional regulation occurs on stimulation by a variety of proinflammatory cytokines, growth factors, and hormones, as well as by interactions between cells or between cells and their surrounding matrix.^14^ MMPs are synthesized as precursor zymogens and are posttranslationally modified and folded within the endoplasmic reticulum (ER) before extracellular export or transport to the plasma membrane. Their activation is dependent on sequential proteolysis of the propeptide that blocks the active site and is regulated by a number of factors, including plasmin, MMP intermediates, and other active MMP family members.^15^ Furthermore, MMP activity can be modulated by exogenous inhibitors, such as α_2_-macroglobulin and a group of tissue inhibitors of metalloproteinases (TIMPs).^16^ The requirement for proteolytic cleavage implies that the conformation of the MMPs is critical for their activation. Hence, posttranslational modifications, such as glycosylation and disulfide bond formation, may serve as novel regulatory pathways under stress conditions that are known to trigger ER stress or the ER unfolded protein response (UPR^ER^). All three UPR^ER^ signaling pathways PKR-like ER kinase (PERK), activating transcription factor 6 (ATF6), and inositol-requiring enzyme 1 (IRE1), can regulate gene expression directly through their downstream transcriptional factors ATF4/C/EBP homologous protein (CHOP), cleaved ATF6, and spliced X-box binding protein 1 (XBP1), respectively.^17^ For example, we have demonstrated that expression of placental growth factor is mediated through ATF4 and ATF6β signaling in placenta of early-onset preeclampsia.^18^

Proinflammatory cytokines have been demonstrated to suppress trophoblast migration,^8^ invasion,^19^ and integration,^20^ resulting in deficient spiral artery remodeling.^21^, ^22^, ^23^ The major source of proinflammatory cytokines in the decidua is the immune cells, of which approximately 70% are decidual natural killer cells and approximately 20% are macrophages.^24^ Decidual natural killer cells have a unique phenotype and properties compared with their peripheral blood counterparts and secrete cytokines and other soluble factors to modulate implantation, placental function, and ultimately fetal development. Aberrant behavior of these cells has been suggested to contribute to the pathogenesis of preeclampsia.^25^, ^26^, ^27^, ^28^, ^29^, ^30^ However, the mechanisms by which these cytokines inhibit trophoblast invasion remain unknown.^31^ Coincidently, proinflammatory cytokines also induce ER stress in other mammalian cell types.^32^, ^33^, ^34^ Therefore, we investigated the potential role of ER stress in the regulation of trophoblast MMP-2 activity, thereby modulating EVT invasion during early pregnancy.

The potential existence of ER stress was first examined in EVTs in placental bed biopsy specimens obtained from pregnancies complicated by early-onset preeclampsia. It was then tested whether ER stress can modulate MMP-2 activity before elucidating the role of proinflammatory cytokines in the induction of ER stress and suppression of MMP-2 activity in extravillous-like trophoblastic cells. Finally, the molecular mechanisms by which the UPR^ER^ pathways may regulate MMP-2 activity were explored.

## Materials and Methods

### Immunohistochemistry

The ethical approval, the criteria diagnosis of early-onset preeclampsia, and the procedures for the placental bed biopsies were described previously.^35^ Immunohistochemistry of cytokeratin 7 (CK7) and heat shock 70-kDa protein 5 (HSPA5) (alias 78-kDa glucose regulated protein) was performed on paraffin-embedded placental villous and placental bed sections (5 μm) using antibodies to CK7 (Dako, Agilent Technologies LDA UK Ltd., Stockport, UK) and HSPA5 (Abcam, Cambridge, UK).

### Cell Cultures

The human choriocarcinoma cell line JEG-3 and the HTR8/SVneo trophoblast cell line were cultured in RPMI 1640 medium (Invitrogen Ltd, Paisley, UK) that contained 10% heat-inactivated fetal bovine serum (Invitrogen), 100 U/mL of penicillin, and 100 μg/mL of streptomycin at 37°C in 5% carbon dioxide atmosphere. Both cell lines express markers of EVT.^36^ On reaching confluence, cells were dissociated from the culture flask using 0.05% Trypsin-EDTA (Invitrogen) for subculture or further experiments.

For experiments, JEG-3 and HTR8/SVneo cells were seeded at 1.25 × 10^5^ cells/mL and 2 × 10^5^ cells/mL, respectively, in 2 mL of culture medium in a six-well plate for 48 hours before treatment. Cells were rinsed with serum-free medium twice before application of cytokines or drugs in 1 mL of serum-free medium for 24 hours. All cytokines were used at 50 ng/mL. For JEG-3 cells, tunicamycin was used at 0.31, 0.62, 1.25, and 2.50 μg/mL for dose-response study and thapsigargin was at 100 nmol/L. For HTR8/SVneo cells, tunicamycin and thapsigargin were used at 78 ng/mL and 125 nmol/L, respectively. GSK2606414 was used at 100 nmol/L and purchased from Generon (Slough, UK). All other drugs, chemicals, and cytokines were purchased from Sigma-Aldrich Company Ltd (Dorset, UK).

### Immunofluorescence

After treatment, cells were fixed with 4% paraformaldehyde in phosphate-buffered saline for 20 minutes. After washing, the cells were incubated with blocking buffer that contained 1% bovine serum albumin (Sigma-Aldrich) and 0.1% saponin (Sigma-Aldrich) in phosphate-buffered saline for 30 minutes before incubating with primary antibody against ATF4 (New England Biolabs, Hitchin, UK) for overnight at room temperature. After washing, cells were incubated with secondary antibody conjugated with fluorescein Alexa 488 (Vector Laboratories, Peterborough, UK) for 1 hour followed by staining in 5 μg/mL of Hoechst 33342 (Sigma-Aldrich) nuclear dye staining for 10 minutes. Images were taken by EVOS FL Color Imaging system (Life Technologies) at ×200 magnification.

### *ATF4* Gene Knockdown

Expression of *ATF4* was suppressed by RNA interference using Lipofactamine RNAiMax (Invitrogen) as described by the manufacturer. In brief, JEG-3 cells (2.5 × 10^5^) were plated in a six-well plate. The day before reaching approximately 30% to 40% confluence, they were transfected with 30 pmol of siRNA molecules targeted against human *ATF4* (SAS1_Hs02_00332313; Sigma-Aldrich) or control siRNA molecules (SIC001; Sigma-Aldrich) using 9 μL of Lipofactamine RNAiMax (Invitrogen) in 300 μL of OptiMEM medium (Invitrogen) for 48 hours. The wells were rinsed with serum-free medium and incubated with 1 mL of serum-free medium that contained 100 nmol/L of thapsigargin (Sigma-Aldrich) for 24 hours.

### Protein Sample Preparation and Western Blot

Protein isolation and Western blotting analysis were performed as previously described.^37^ Primary antibodies for ATF4, eukaryotic translation initiation factor 2A (EIF2A), phosphorylated EIF2A(Ser51) [p-EIF2A(Ser51)], and MMP-2 were from Cell Signaling Technology (New England Biolabs, Hitchin, UK), HSPA5 (alias GRP78) from Transduction Lab (BD Biosciences, Wokingham, UK), and TIMP1 from Abcam. The results are shown as follows: Relative Ratio (%) = (Density of Treatment Group/Density of Control) × 100%.

### Zymogen Assay for MMP-2 Activity

MMP-2 activity was determined by gelatin gel zymography. In brief, the conditioned media were concentrated, and equal volumes were used for native gel electrophoresis. After resolving, the gel was incubated in 2.5% Triton X-100 for 1 hour before incubation in buffer that contained 10 mmol/L calcium chloride, 200 mmol/L sodium chloride, and 50 mmol/L Tris hydrochloride overnight at 37°C. The gel was stained with PAGE blue (ThermoFisher Scientific, Waltham, MA) overnight before destaining with water.

### Trophoblast Invasion Assay

Invasive potential was determined by the Transwell invasion assay using an 8-μm insert (BD Biosciences, Wokingham, UK). In brief, HTR8/SVneo cells (1 × 10^5^ per well) in serum-free culture medium were placed in the upper chamber, whereas the lower chamber contained culture medium with 10% fetal bovine serum. After 24 hours, the medium and the cells in the upper chamber were discarded. Cells that had invaded through to the undersurface of the membrane were fixed and permeabilized with ice-cold methanol, stained with 0.6 μg/mL of SYTOX-green (S33025, Invitrogen), and visualized with a Litz DM1L microscope (Leica Microsystems, Wetzlar, Germany). The number of cells was quantified using ImageJ software version 1.51h (NIH, Bethesda, MD; *http://imagej.nih.gov/ij*). The results are given as follows: Rate of Invasion (%) = (Invasiveness of Treatment Group/Invasiveness of Control) × 100%.

### Reverse Transcription and Quantitative RT-PCR

Total RNA was isolated using Qiagen RNeasy Mini Kits (Qiagen Ltd, Manchester, UK) following the manufacturer's instructions. cDNA synthesis was performed as previously described.^37^ Both TaqMan and SYBR Green were used to quantify gene expression. For TaqMan, the probes were *MMP2* (Hs01548727_m1), *GAPDH* (Hs99999905_m1), and *18S* (Hs99999901_s1) using TaqMan Gene Expression Assays (ThermoFisher Scientific). For SYBR Green, the primers were *ATF4* (sense: 5′-GACGGAGCGCTTTCCTCTT-3′; antisense: 5′-TCCACAAAATGGACGCTCAC-3′); *18S* (sense: 5′-GTAACCCGTTGAACCCCATT-3′; antisense: 5′-CCATCCAATCGGTAGTAGCG-3′); *TBP* (sense: 5′-GTGGGGAGCTGTGATGTGA-3′; antisense: 5′-AATAAGGAGAACAATTCTGGTTTG-3′) and were analyzed by SYBR Green JumpStart kits (Sigma-Aldrich). Gene expression levels were determined using the threshold cycle (2^−ΔΔCT^) method with reference to the endogenous controls of either *18S* and *GAPDH* or *18S* and *TBP*. The results are presented as relative expression.

### Statistical Analysis

All values are expressed as means ± SEM. Differences between study groups were analyzed by the one-way analysis of variance test or paired two-tailed nonparametric Friedman test when appropriate. Correlation analysis was conducted using the Pearson test. *P* < 0.05 was considered significant. Statistical analysis was performed using the SPSS version 21.0 (IBM Corp, Armonk, NY) or Prism GraphPad version 6.0 (GraphPad Sofware Inc, San Diego, CA).

## Results

### Existence of ER Stress in the EVT in Placental Bed Samples from Preeclamptic Pregnancies

**Figure 1:**
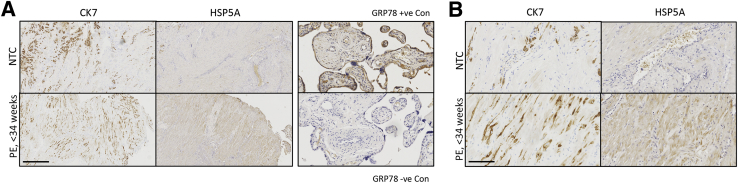
Endoplasmic reticulum (ER) stress is present in extravillous trophoblasts of placental bed samples from patients with preeclampsia (PE). Immunohistochemistry for the trophoblast marker cytokeratin 7 (CK7) and the ER stress biomarker heat shock 70-kDa protein 5 (HSPA5) [alias 78-kDa glucose regulated protein (GRP78)] was performed on serial sections from placental bed samples from both patients with PE and normotensive controls (NTCs) Placental villous tissue was used as a positive control for HSPA5 immunostaining. Scale bars: 500 μm (**A**); 100 μm (**B**). Original magnification: ×50 (**A**); ×200 (**B**).

The study by Lian et al^38^ demonstrated an increase of ER stress in the decidual tissues of preeclamptic patients. Therefore, immunohistochemistry was performed for HSPA5 and CK7 on serial sections to confirm the existence of high level of ER stress in EVTs within placental bed samples collected from preeclamptic and normotensive pregnancies after caesarean delivery. There was no statistically significant difference in maternal age and maternal body mass index between the two groups (Table 1), but there were significant (*P* < 0.001) differences in systolic and diastolic blood pressure, gestational age, and birth weight. There were many CK7-positive EVTs in both preeclamptic and normotensive control pregnancies (Figure 1A). Increased HSPA5 staining in the preeclamptic compared with the normotensive samples confirmed a higher degree of ER stress in those cases (Figure 1B). ER stress was not restricted solely to the EVTs, and decidual cells also had strong immunoreactivity, as in the study by Lian et al.^38^ It was also noteworthy that the staining was not even across the entire preeclamptic placental bed, indicating the existence of regional variations (data not shown).

**Table 1:** Clinical Characteristics of Normotensive Controls and Patients with Early-Onset Preeclampsia

Characteristic	Normotensive controls (*n* = 9)	Patients with early-onset preeclampsia (*n* = 7)	*P*
Maternal age, years	33 ± 1.6	28.9 ± 1.8	0.148
Body mass index	24.2 ± 1.7	27.8 ± 2.2	0.204
Systolic blood pressure, mm Hg	122.6 ± 2.4	187.9 ± 4.2	0.001
Diastolic blood pressure, mm Hg	76.7 ± 1.9	113 ± 2.5	0.001
Gestational age, days	274.8 ± 0.8	208.4 ± 5.1	0.001
Birth weight, g	3635.2 ± 111.4	1037.9 ± 115.3	0.001

Data are expressed as means ± SD unless otherwise indicated.

### Induction of ER Stress Suppresses MMP-2 Expression, Secretion, and Activity in Trophoblasts

**Figure 2:**
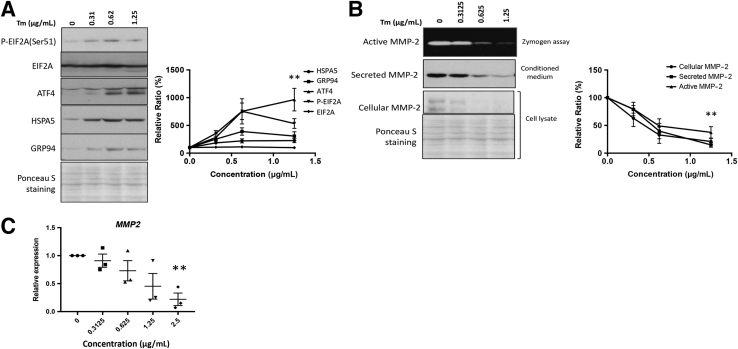
Endoplasmic reticulum (ER) stress reduces matrix metallopeptidase (MMP)-2 expression, secretion, and activity. JEG-3 cells were subjected to a dose-response study with tunicamycin (Tm) at 0.31, 0.62, and 1.25 μg/mL for 24 hours. **A:** Levels of phosphorylated eukaryotic translation initiation factor 2A (p-EIF2A), EIF2A, activating transcription factor 4 (ATF4), heat shock 70-kDa protein 5 (HSPA5), and glucose regulated protein 94 (GRP94) were measured by Western blotting. **B:** MMP-2 expression and secretion were determined by Western blot, whereas activity was analyzed by gelatin gel zymography. **C:** Transcript levels of *MMP2* were quantified by quantitative RT-PCR. Data are expressed as means ± SEM. *n* = 3. ^∗∗^*P* < 0.01 versus 0 μg/mL.

Invasion of EVTs into the endometrium and inner one-third of the myometrium is a crucial stage in human placentation and is mainly mediated by MMP-2 in the first trimester.^12^, ^13^, ^39^ We therefore investigated whether ER stress can directly alter MMP-2 activity using the ER stress inducer tunicamycin and extravillous-like trophoblast JEG-3 cells. Tunicamycin triggered a dose-dependent increase in severity of ER stress in JEG-3 cells as demonstrated by the biomarkers p-EIF2A, ATF4, HSPA5, and GRP94 (Figure 2A), which were increased by 533% ± 88%, 965% ± 204%, 288% ± 41%, and 302% ± 141%, respectively, at 1.25 μg/mL (Figure 2A). Crucially, tunicamycin treatment also reduced the cellular levels, secretion, and activity of MMP-2 in a dose-dependent manner (Figure 2B). At 1.25 μg/mL, these were suppressed by 21.0% ± 5.7%, 15.2% ± 4.1%, and 37.5% ± 10.3%, respectively (Figure 2B). To investigate whether the inhibition of cellular MMP-2 operates at the transcriptional or translational level, *MMP2* transcripts were assayed by quantitative RT-PCR. A dose-dependent reduction was observed on tunicamycin treatment (Figure 2C).

### Induction of ER Stress Inhibits Trophoblast Invasion

**Figure 3:**
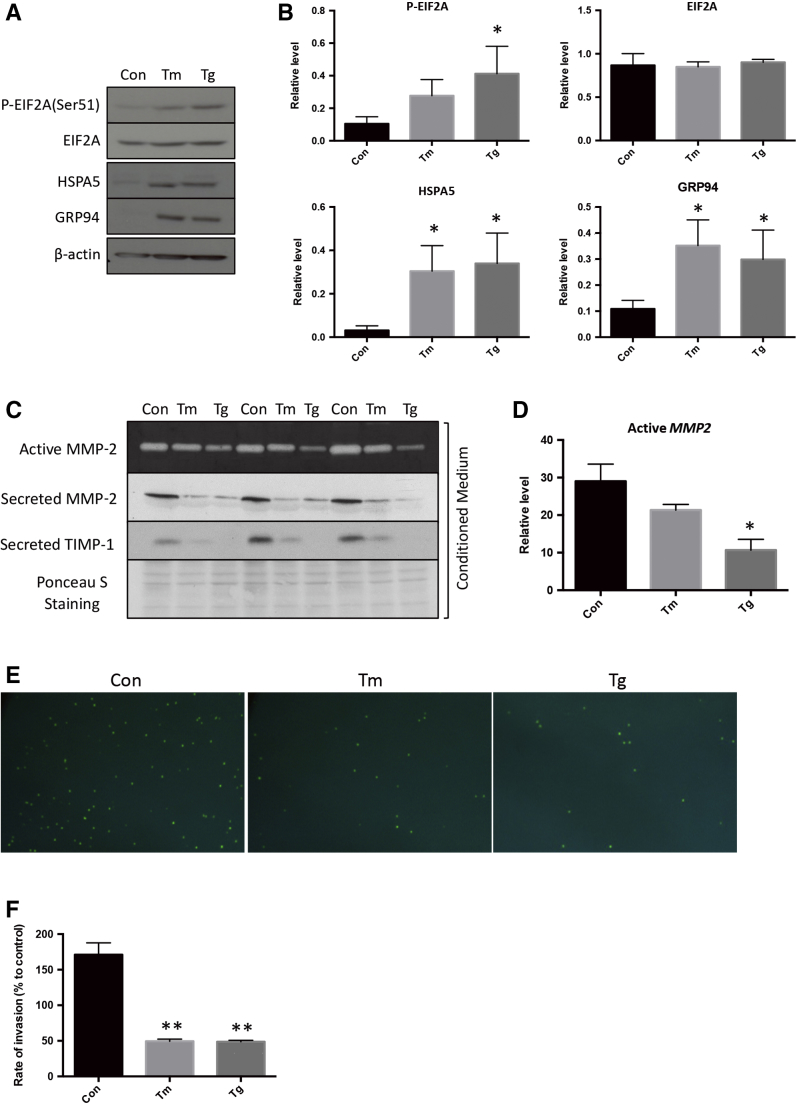
Endoplasmic reticulum (ER) stress mediates down-regulation of matrix metallopeptidase (MMP)-2 activity and is associated with a reduction in trophoblast invasiveness. HTR8/SVneo were treated with 0.078 μg/mL of tunicamycin (Tm) or 125 nmol/L thapsigargin (Tg) for 24 hours. **A:** Levels of phosphorylated eukaryotic translation initiation factor 2A (p-EIF2A), EIF2A, heat shock 70-kDa protein 5 (HSPA5), and glucose regulated protein 94 (GRP94) were determined by Western blotting. **B:** Densitometry analysis of p-EIF2A, EIF2A, HSPA5, and GRP94. **C:** Levels of MMP-2 and secretion of MMP-2 and tissue inhibitors of metalloproteinase 1 (TIMP1) were determined by Western blotting. MMP-2 activity was measured by gelatin gel zymography. **D:** Densitometry analysis of cellular, secreted, and active *MMP2*. **E:** Invasion of trophoblast cells was determined by Transwell invasion assay with SYTOX-green fluorescence staining. **F:** Quantification of the rate of invasion. Data are expressed as means ± SEM. *n* = 3. ^∗^*P* < 0.05, ^∗∗^*P* < 0.01 versus control. Original magnification, ×200 (**E**). Con, control.

To eliminate drug- and cell-specific effects, another trophoblast cell line, the first trimester immortalized HTR8/SVneo cell, and an additional ER stress inducer, thapsigargin, were introduced. Both thapsigargin and tunicamycin stimulated higher levels of p-EIF2A, HSPA5, and GRP94 (Figure 3, A and B) and reduced secretion and activity of MMP-2 (Figure 3, C and D). In addition, the secreted form of TIMP1, an extracellular inhibitor of MMP-2, was also reduced, indicating that the loss of MMP-2 activity is unlikely mediated by the TIMP1 regulatory mechanism (Figure 3C). Finally, a Transwell invasion assay was used to demonstrate the effect of ER stress on HTR8/SVneo cell invasion. Treatment with the ER stress inducers caused a >70% reduction in cells penetrating through the Matrigel compared with controls, confirming reduced invasive capacity (Figure 3, E and F).

### Inhibition of Trophoblast MMP-2 Expression, Secretion, and Activity by Proinflammatory Cytokines Is Accompanied by Induction of ER Stress

**Figure 4:**
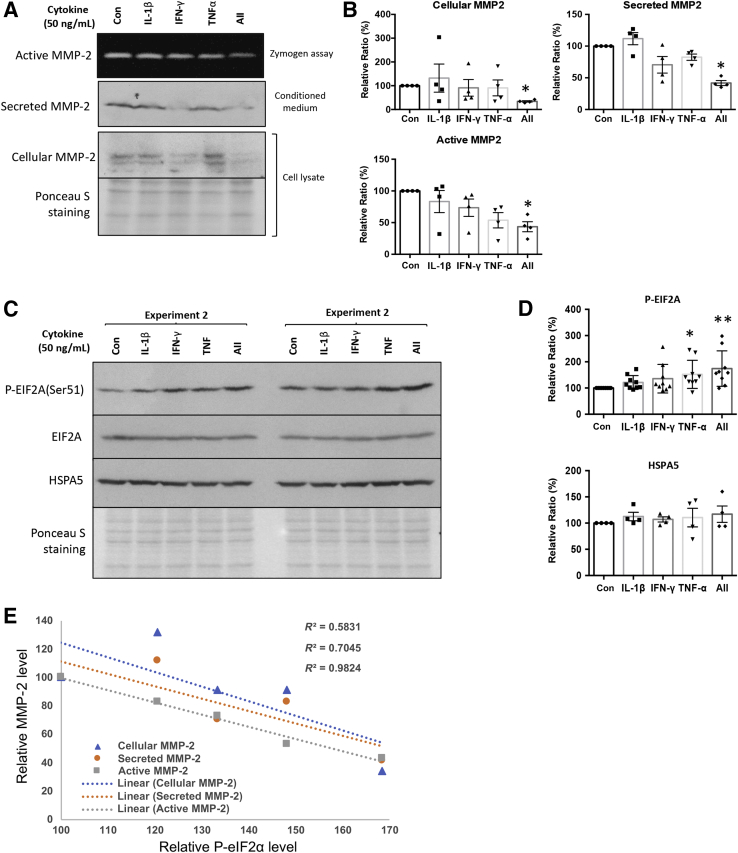
Proinflammatory cytokines inhibit trophoblast matrix metallopeptidase (MMP)-2 levels, secretion, and activity and induce endoplasmic reticulum (ER) stress. JEG-3 cells were treated with interleukin (IL)-1β, interferon (IFN)-γ, tumor necrosis factor (TNF)-α, and all cytokines for 24 hours. **A:** MMP-2 levels and secretion were determined by Western blotting. MMP-2 activity was analyzed by gelatin gel zymography. **B:** Densitometry of the cellular, secreted, and active MMP-2. **C:** Levels of ER stress biomarkers, P-EIF2A, EIF2A, and HSPA5 were measured by Western blotting. **D:** Densitometry analysis of the phosphorylated eukaryotic translation initiation factor 2A (p-EIF2A), EIF2A, and heat shock 70-kDa protein 5 (HSPA5). **E:** Regression analysis showing the correlation among MMP-2 level, secretion, and activity with p-EIF2A level. Data are expressed as means ± SEM. *n* = 4 (**A** and **B**); *n* = 4 to 9 (**C** and **D**). ^∗^*P* < 0.05, ^∗∗^*P* < 0.01 versus control. Con, control.

Proinflammatory cytokines, which can result from infection or systemic inflammation, are known to inhibit trophoblast invasion. They also trigger ER stress in other human cells, as well as in villous-like trophoblast BeWo cells.^33^, ^40^, ^41^ Therefore, three proinflammatory cytokines, IL-1β, tumor necrosis factor (TNF)-α, and interferon (IFN)-γ were administered to JEG-3 cells. Western blotting analysis revealed that cells treated with all three cytokines had significantly reduced levels of cellular MMP-2 (33.7% ± 2.9%), secretion (41.8% ± 4.0%), and activity (43.5% ± 8.0%) compared with the control group (Figure 4, A and B). By contrast, JEG-3 cells treated with individual cytokines had only very subtle or no effects (Figure 4B).

Next, it was investigated whether the proinflammatory cytokines were able to induce ER stress in JEG-3 cells. Treatment with TNF-α or all cytokines together increased levels of p-EIF2A, a marker of ER stress, significantly by 148.2% ± 16.5% and 168.4% ± 21.0%, respectively, compared with controls (Figure 4, C and D). The ER chaperone HSPA5 remained unchanged, indicating only low-grade ER stress (Figure 4, C and D). Correlation analysis between p-EIF2A and MMP-2 (cellular, secreted, and active forms) had very strong inverse associations (*R*^2^ = 0.58, 0.70, and 0.98, respectively) (Figure 4E). These results indicated potential regulation of MMP-2 by the PERK-EIF2A arm of the UPR pathway.

### ATF4 and EIF2A Negatively Regulate *MMP2* Transcription and Translation in Response to ER Stress

**Figure 5:**
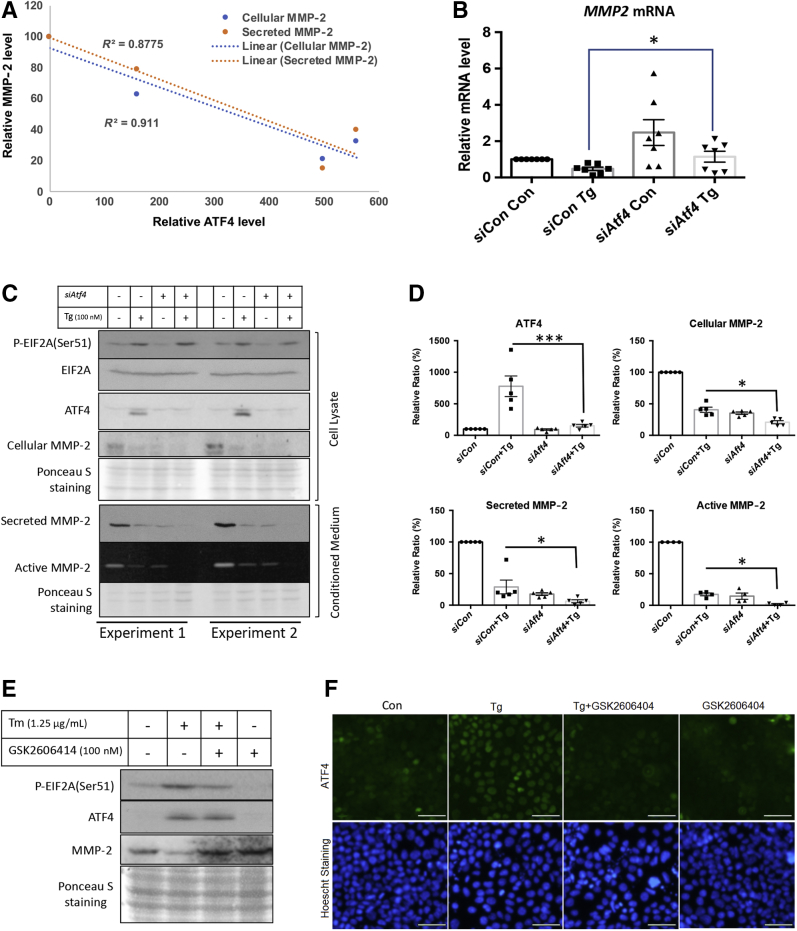
Endoplasmic reticulum (ER) stress–mediated suppression of matrix metallopeptidase (MMP)-2 expression is via the eukaryotic translation initiation factor 2A (EIF2A)–activating transcription factor 4 (ATF4) pathway. JEG-3 cells were treated with *ATF4* siRNA for 48 hours followed by 100 nmol/L of thapsigargin (Tg) treatment for 24 hours. **A:** Regression analysis showing the correlation between *MMP2* expression and secretion with ATF4 level. **B:** Quantitative RT-PCR reveals up-regulation of *MMP2* mRNA in *siATF4*-transfected cells with or without thapsigargin treatment. **C:** Levels of phosphorylated eukaryotic translation initiation factor 2A (p-EIF2A), EIF2A, activating transcription factor 4 (ATF4), cellular MMP-2, and secreted MMP-2 in *siATF4*-transfected cells were determined by Western blotting. MMP-2 activity was determined by gelatin gel zymography. **D:** Densitometry of the level of ATF4 and cellular, secreted, and active MMP-2. **E:** MMP-2 protein under ER stress was measured in the presence or absence of PERK inhibitor GSK2606414. JEG-3 cells were treated with ER stress inducer and/or GSK2606414 (100 nmol/L) before Western blotting for MMP-2, ATF4, p-EIF2A, and EIF2A. **F:** Immunofluorescence was used to show the localization of ATF4 protein under ER stress in the presence or absence of GSK2606414. Data are expressed as means ± SEM. *n* = 3 to 5. ^∗^*P* < 0.05, ^∗∗∗^*P* < 0.001. Scale bars = 50 μm. Original magnification: ×200. Con, control.

ATF4 is a transcription factor downstream of the PERK-EIF2A arm of the UPR signaling pathway. Therefore, its potential role in regulating *MMP2* transcription was investigated. A negative correlation (*R*^2^ = 0.88 and 0.91) between the levels of ATF4 and cellular or secretory MMP-2 was observed in JEG-3 cells (Figure 5A). These results suggested potential regulation of *MMP2* transcription by ATF4, and therefore siRNA was used to knockdown *ATF4* transcripts before treatment with thapsigargin.

*siATF4* treatment abolished the increase in ATF4 induced by thapsigargin (Figure 5, C and D). As expected, knockdown of the *ATF4* gene induced a 2.2-fold increase of *MMP2* mRNA level in *siAFT4*-treated cells in the presence or absence of thapsigargin (Figure 5B). However, the increased *MMP2* transcripts failed to translate into protein because the cellular MMP-2 level was still reduced by 65% in *siATF4*-transfected cells. Elevation of *MMP2* transcripts also failed to restore cellular MMP-2 protein levels on treatment with thapsigargin, which still had a 49% reduction in the *siATF4*-transfected cells compared with *siCon*-transfected cells (Figure 5, C and D). PERK-mediated phosphorylation of EIF2A causes attenuation of nonessential protein translation.^42^ To investigate the potential translational regulation of MMP-2 by PERK-EIF2A, a PERK inhibitor, GSK2606414, was added in the presence and absence of the ER stress inducers tunicamycin or thapsigargin. Application of GSK2606414 reduced ER stress–mediated phosphorylation of EIF2A and restored the MMP-2 protein level (Figure 5E). However, ATF4 was maintained at a high level under the treatment. Nuclear localization is crucial for transcriptional regulation. Therefore, ATF4 cellular localization was examined by immunocytochemistry. Indeed, ATF4 nuclear localization was largely absent in the presence of GSK2606414, whereas there was clearly nuclear staining of ATF4 in untreated cells on ER stress (Figure 5F). These results strongly suggest that the expression of MMP-2 is regulated both transcriptionally and translationally by the PERK-EIF2A-ATF4 pathway in response to ER stress.

## Discussion

ER stress has been demonstrated in both the placenta and decidua in cases of idiopathic FGR and early-onset preeclampsia.^38^, ^43^ In the placenta, the stress is likely a consequence of hypoxia-reperfusion injury triggered by insufficient remodeling of the spiral arteries. However, the trigger for the decidual stress is unknown, and its effect on pregnancy outcome has not been explored. In this study, proinflammatory cytokines were identified as a potential source of decidual ER stress, and the inhibitory role ER stress exerts on trophoblast invasion through modulation of MMP-2 activity was demonstrated. Furthermore, our results elucidated that the PERK arm of UPR signaling in ER can directly regulate MMP-2 at both the translational and transcriptional levels by PERK-EIF2A and ATF4, respectively. Overall, these results provide new insights into the molecular pathological mechanisms that may underpin cases of reduced trophoblast invasion, and hence spiral artery remodeling, in pregnancy complications.

Several pregestational pathological conditions result in aberrant increases of proinflammatory cytokines within the uterine cavity and are associated with an increased risk of preeclampsia. For example, infection with *Chlamydia trachomatis* increases levels of IFN-γ in cervical secretions,^44^ the secretion of IL-1β and TNF-α in dendritic cells,^45^ and the risk of preeclampsia.^46^ Elevated levels of uterine proinflammatory cytokines likely induce ER stress in the invading EVTs, in a similar fashion to the way that injection of IL-1β and IL-6 induces ER stress in pancreatic Islet cells.^47^ Compromise of MMP activity will inhibit invasion into the deeper regions of the endometrium and myometrium, and the clinical outcome will be dependent on the severity of the subsequent deficit in arterial remodeling. Milder cases will result in preeclampsia and/or FGR, whereas severe cases will end in miscarriage. The severity of ER stress determines trophoblast cell fate; at low levels it reduces cell proliferation, whereas at high levels it induces apoptosis.^43^

The literature regarding regulation of trophoblast invasion by proinflammatory cytokines is contentious. Although many studies have revealed their inhibitory role, there is also evidence that the same cytokines may promote trophoblast migration and invasion.^22^, ^23^, ^48^, ^49^ In normal pregnancy, IL-1β (1 to 10 ng/mL) up-regulates the proteases MMPs and urokinase-type plasminogen activator systems to promote trophoblast motility,^48^, ^49^ whereas decidual natural killer cell–derived IFN-γ is necessary for spiral artery remodeling and placental formation.^22^, ^23^ On the other hand, inhibition of trophoblast invasion by IFN-γ associated with reduced secretion of MMP-2 has been reported.^19^ In addition, TNF-α inhibits trophoblast migration^8^ and integration into maternal endothelial cellular networks, which also involves the inhibition of MMP-2.^20^ In a rat model, TNF-α is causally linked to deficient trophoblast invasion and spiral artery remodeling, leading to features of preeclampsia and FGR.^21^ These results reveal the complexity of the regulation of trophoblast invasion by proinflammatory cytokines. Although the mechanisms behind these opposite roles are unknown, the concentration of the cytokines, their spatial and temporal profiles, their sources or origins (immune cells or endometrial cells), and their interactions with other cytokines may explain the differences.^24^, ^50^ Changes in proinflammatory cytokine profiles may also alter the interactions between decidual natural killer cells and trophoblast cells, thereby modulating the invasion process.^51^ The local milieu is therefore likely to be critical, but mimicking the precise conditions within the decidua *in vivo* is difficult in reductionist experimental situations. For example, TNF-α and IFN-γ, but not IL-1β, inhibited MMP-2 activity when administered individually, whereas a mixture of all three cytokines produced a synergistic effect (Figure 4). Crucially, similar effects were also observed in their capacity to activate phosphorylation of EIF2A, the PERK arm of UPR pathway in ER.

Proinflammatory cytokines induce ER stress in many mammalian cell systems.^32^, ^33^, ^34^ Our results demonstrate that these cytokines also trigger ER stress in trophoblast JEG-3 cells. However, the severity of stress is likely low grade because only the PERK-EIF2A arm of the UPR^ER^ pathway was activated. Similar low-grade ER stress was observed in the trophoblast cells of the human placenta after pregnancy at high altitude^52^ and in the mouse placenta on hypoxic challenge.^53^ It has been suggested that the severity of ER stress induced by the proinflammatory cytokines can be cell type specific and also species specific.^33^ The mechanisms by which they activate UPR pathways in ER are unclear, but several studies suggest both direct activation and indirect activation mediated by nitric oxide or perturbation of calcium homeostasis.^54^, ^55^ A mixture of IL-1β, TNF-α, and IFN-γ induces splicing of XBP1, a downstream effector of the IRE1α arm of the UPR^ER^ pathway, and phosphorylation of Eif2A in mouse islet and MIN6 cells independent of nitric oxide production.^54^ Conversely, a combination of IL-1β and IFN-γ facilitates ER Ca^2+^ depletion mediated through inhibition of the sarcoplasmic reticulum Ca^2+^ ATPase (SERCA2B), as well as production of nitric oxide in pancreatic β cells.^55^ The activation of IRE1α by these cytokines is likely to be transient. The study by Brozzi et al^33^ found that IL-1β and IFN-γ gradually facilitate IRE1α activation, which peaks at 16 hours before decreasing. This finding may explain why only activation of PERK-EIF2A was observed in this study where the incubation time was limited to 24 hours. Finally, the IRE1α signaling pathway has been linked to cellular inflammatory response mechanisms through the JNK and NF-κB pathways, thereby possibly providing a positive feedback loop.^56^

Other stressors closely linked to the preeclampsia may also act through the same pathways, for example, high levels of maternal endothelin (ET)-1 or elevated plasma concentrations of homocysteine.^57^, ^58^, ^59^ We found that ET-1 down-regulates MMP-14 and -15 expression in first trimester trophoblast cells,^60^ and both ET-1 and homocysteine induce ER stress in trophoblast and other cell types.^61^, ^62^ Therefore, the finding of ER stress–regulated *MMP2* expression may provide an additional mechanistic explanation for the actions of ET-1 and homocysteine in the development of pregnancy complications.

These results elucidated the coexistence of both transcriptional and translational regulation of MMP-2 by ATF4 and EIF2A, respectively, under ER stress. This finding is supported by the siRNA-mediated knockdown of the *ATF4* gene and GSK2606414-mediated suppression of phosphorylation of EIF2A in restoration of *MMP2* transcript and protein levels, respectively (Figure 5, E and F). Interestingly, ATF4 nuclear localization was blocked by GSK2606414 treatment under ER stress. Although the mechanism behind this failure is unknown, recent studies have revealed roles for posttranslational modifications in determination of ATF4 protein stability, nuclear localization, and transcriptional activity.^63^ Nevertheless, without nuclear translocation, the inhibitory role of ATF4 in *MMP2* transcription is minimal, thereby facilitating both transcription and translation of MMP-2 in the presence of GSK2606414. A combination of both transcriptional and translational regulation of MMP-2 ensures no cells invade into or towards an unfavorable environment.

Finally, these results are consistent with the study by Lian et al,^38^ which indicate that decidual ER stress is increased in pregnancies complicated by FGR and preeclampsia via up-regulation of the PERK/EIF2A signaling mechanism. We recognize that use of placental bed samples collected after delivery at term was a limitation in this study. Ideally, placental bed samples from the first trimester are the most appropriate, but these are impossible to obtain for ethical and technical reasons. The use of trophoblast cell lines is also not ideal, but primary trophoblast cells demonstrate high levels of ER stress induced during the isolation procedure (H.W. Yung, unpublished data). This stress would mask the low-grade ER stress induced by the treatment with proinflammatory cytokines and confound the experiments.

To conclude, although there may be other mechanistic links for the inhibition of trophoblast invasion by proinflammatory cytokines, this study provides the first evidence that ER stress plays a role through the PERK-EIF2A-ATF4 arm of the UPR pathway. The results further elucidate a potential pathophysiologic bridge across inflammation, ER stress, and suboptimal trophoblast invasion in pregnancy, explaining why women with uterine or metabolic inflammation have an increased risk of developing preeclampsia. These new insights highlight potential therapeutic interventions aimed at alleviating ER stress at the fetal-maternal interface, facilitating trophoblast invasion and promoting normal placentation.
